# Supplementary material for: Dynamic expression of tRNA‐derived small RNAs define cellular states
Source: EMBO Rep. 2019 Jun 12;20(7):e47789. doi: 10.15252/embr.201947789 (PMC6607006; doi:10.15252/embr.201947789)
Supplement: Supplementary file 1 — Appendix [file EMBR-20-e47789-s001.pdf]

Table Contents

Appendix Figure S1	2
Appendix Figure S2	3
Appendix Figure S3	7

Appendix Figure S1 **FACSDiva Version 6.1.3**

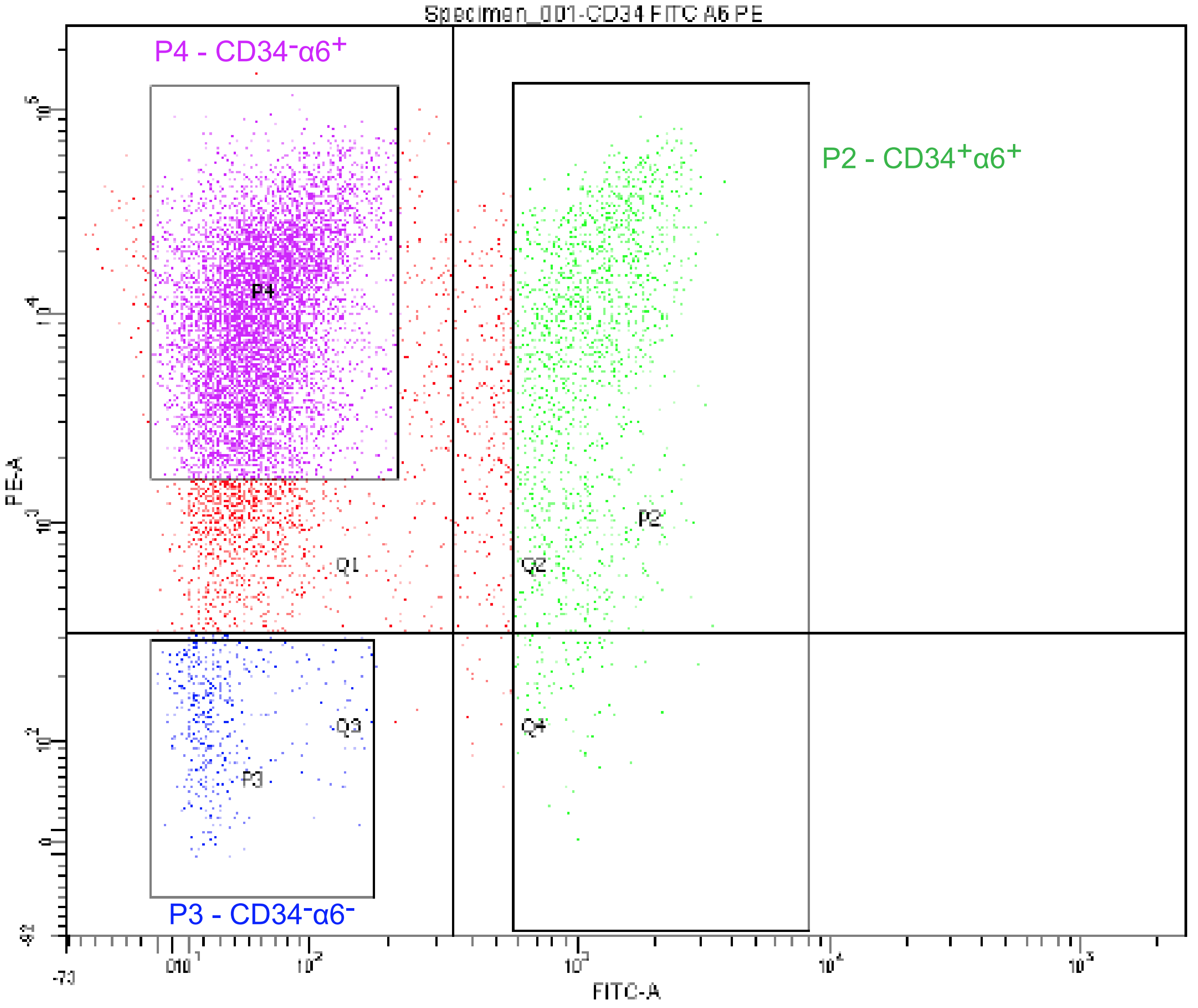

Experiment Name:	190615
Specimen Name:	Specimen_001
Tube Name:	CD34 FITC A6 PE
Record Date:	Jun 19, 2015 5:58:30 PM
$OP:	Administrator
GUID:	fc7a8f2b-3840-49ac-a181-3eff1f4764f2

Population	#Events	%Parent	FITC-A Mean	PE-A Mean
All Events	30,000	####	349	13,095
P1	11,556	38.5	250	12,075
Q1	9,057	78.4	66	12,621
Q2	1,919	16.6	1,136	13,100
Q3	473	4.1	38	147
Q4	107	0.9	892	194
P2	1,750	15.1	1,228	13,403
P3	460	4.0	36	143
P4	7,896	68.3	64	13,968

Appendix Figure S2a

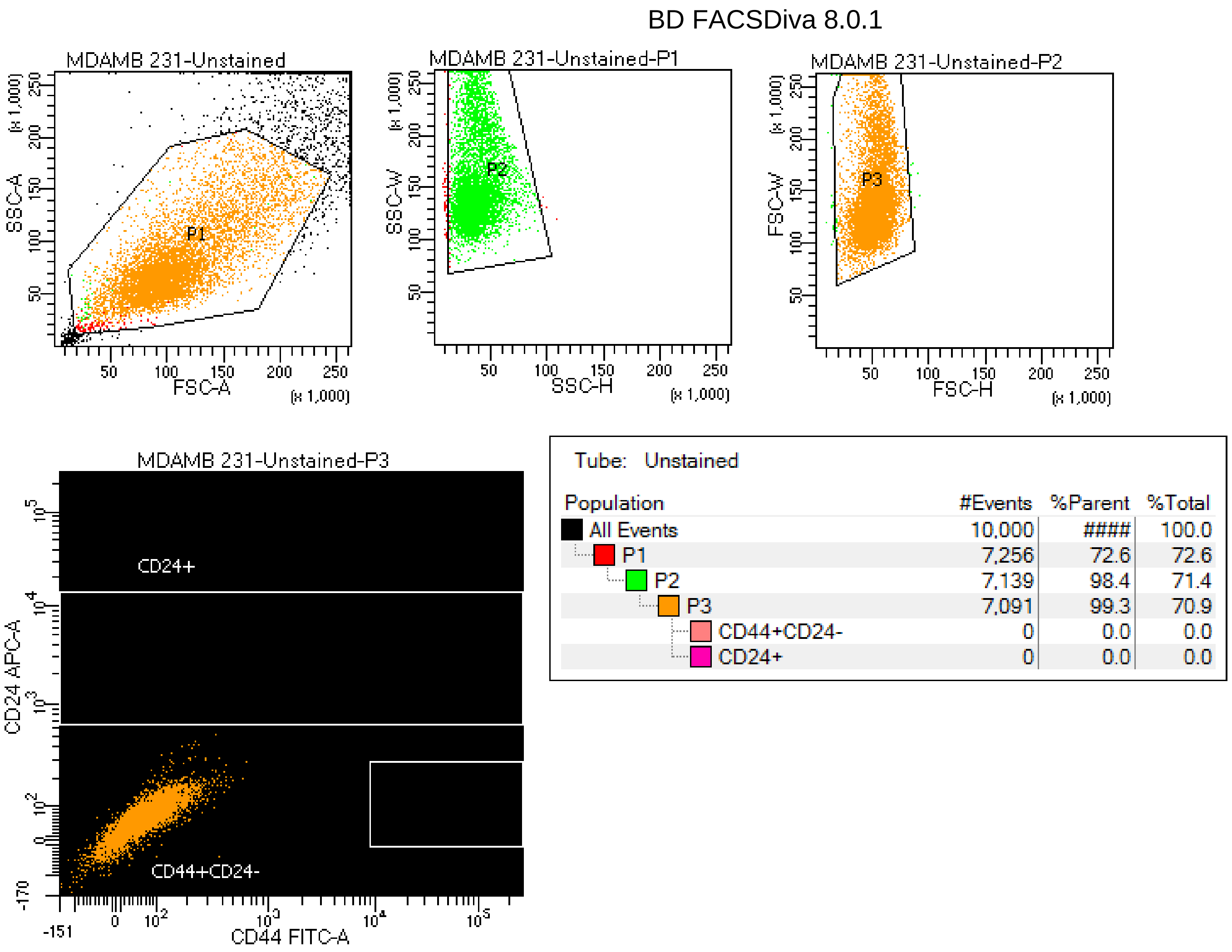

Tube: Unstained

Population	#Events	%Parent	%Total
All Events	10,000	####	100.0
P1	7,256	72.6	72.6
P2	7,139	98.4	71.4
P3	7,091	99.3	70.9
CD44+CD24-	0	0.0	0.0
CD24+	0	0.0	0.0

Appendix Figure S2b

BD FACSDiva 8.0.1

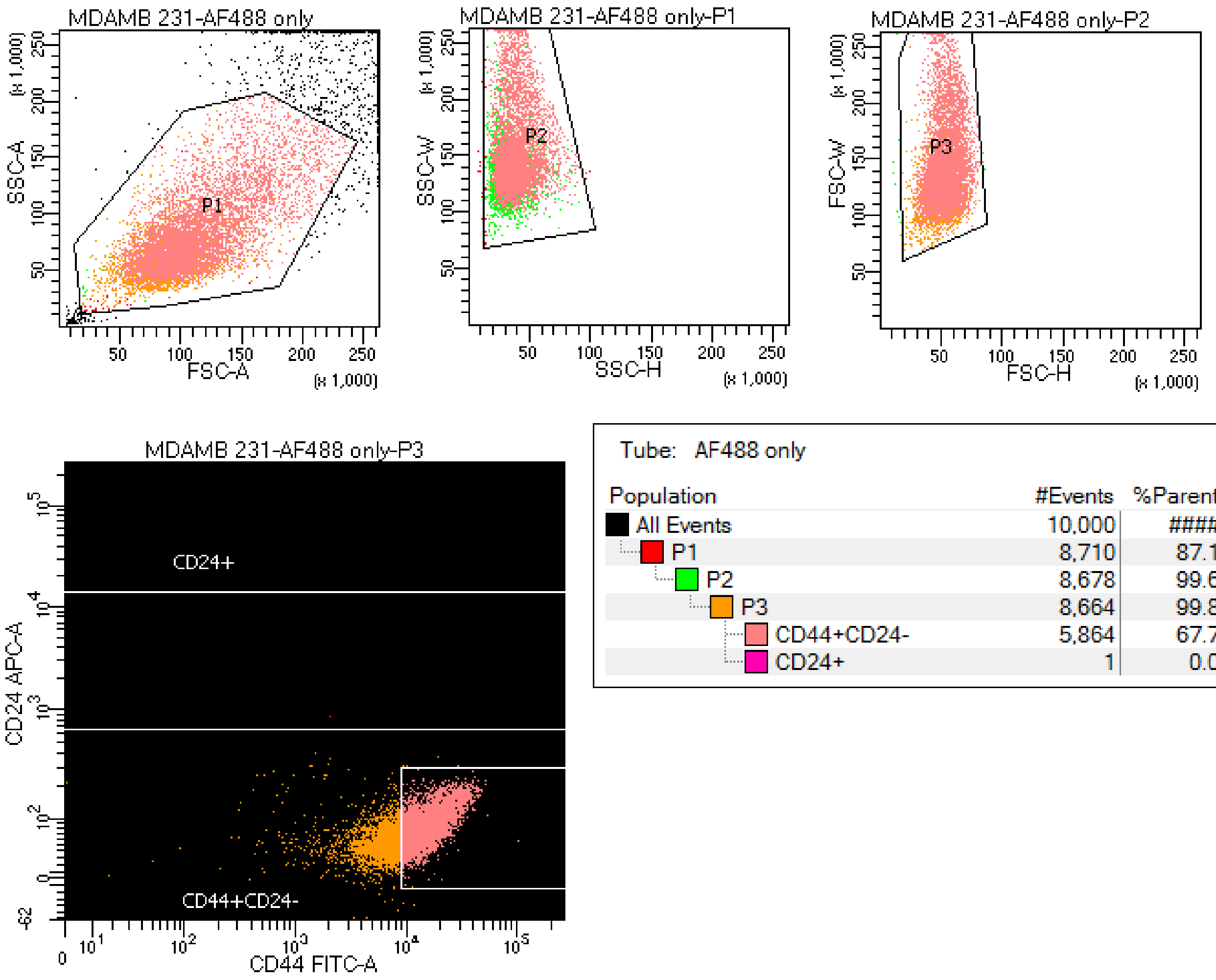

Tube: AF488 only

Population	#Events	%Parent	%Total
All Events	10,000	####	100.0
P1	8,710	87.1	87.1
P2	8,678	99.6	86.8
P3	8,664	99.8	86.6
CD44+CD24-	5,864	67.7	58.6
CD24+	1	0.0	0.0

Appendix Figure S2c

BD FACSDiva 8.0.1

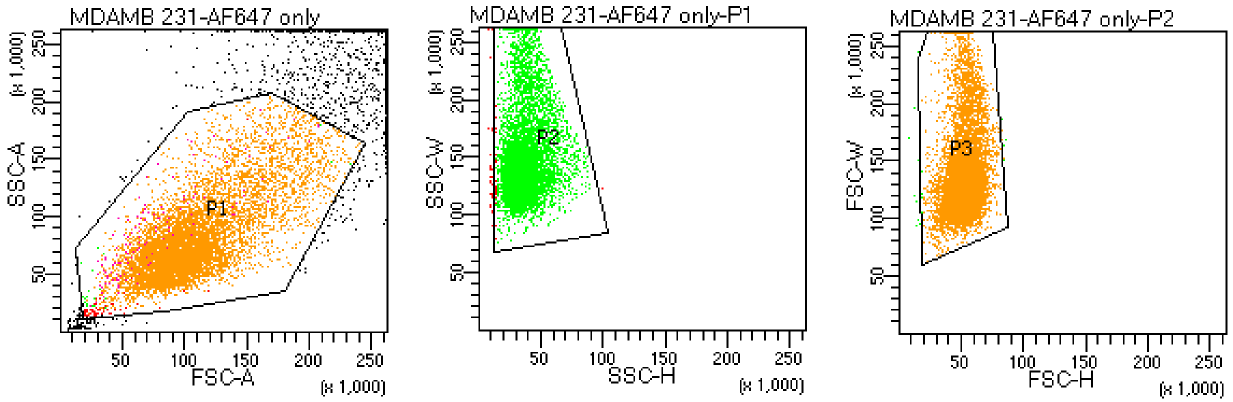

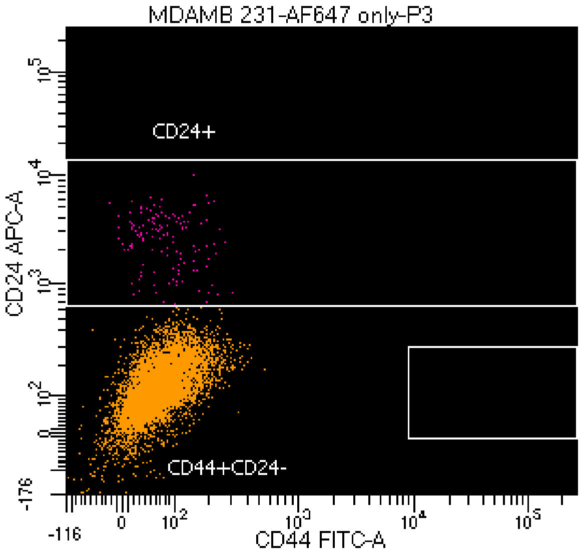

Tube: AF647 only

Population	#Events	%Parent	%Total
All Events	10,000	####	100.0
P1	8,140	81.4	81.4
P2	8,073	99.2	80.7
P3	8,048	99.7	80.5
CD44+CD24-	0	0.0	0.0
CD24+	133	1.7	1.3

Appendix Figure S2d

BD FACSDiva 8.0.1

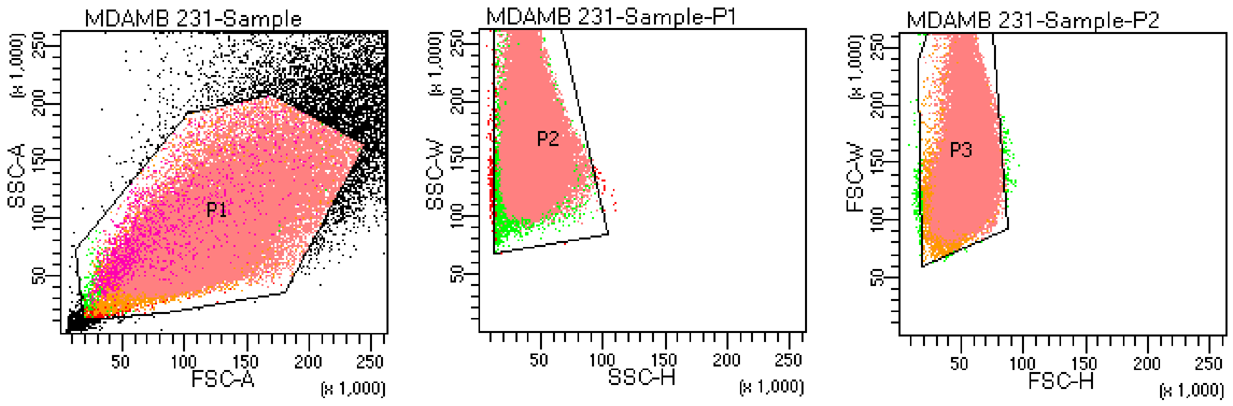

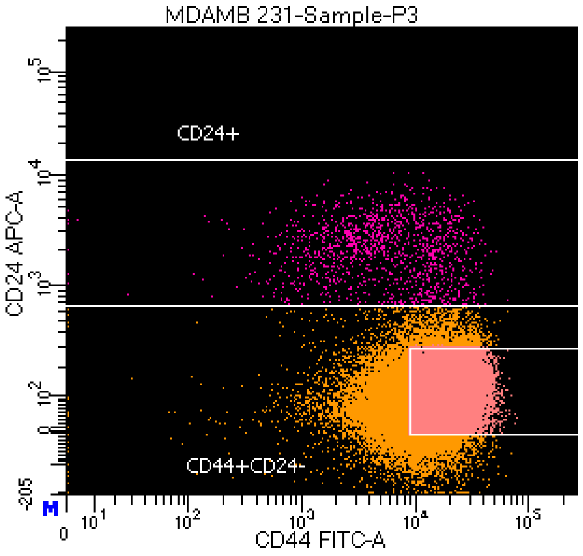

Tube: Sample

Population	#Events	%Parent	%Total
All Events	113,737	####	100.0
P1	101,452	89.2	89.2
P2	101,032	99.6	88.8
P3	100,703	99.7	88.5
CD44+CD24-	75,506	75.0	66.4
CD24+	1,356	1.3	1.2

Appendix Figure S3a

BD FACSDiva 8.0.1

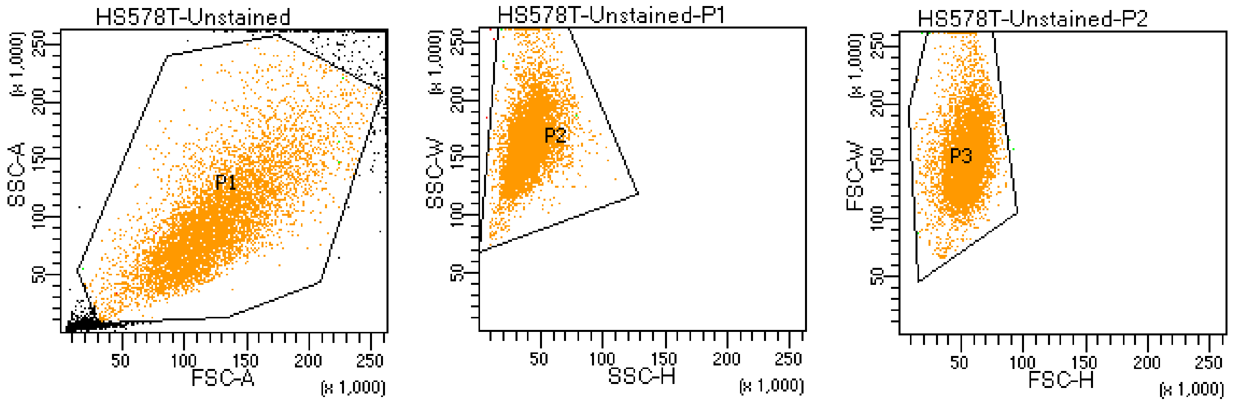

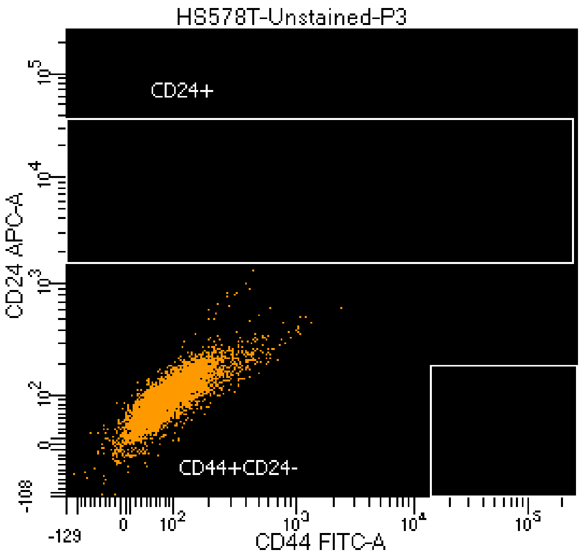

Tube: Unstained

Population	#Events	%Parent	%Total
All Events	10,000	####	100.0
P1	7,357	73.6	73.6
P2	7,350	99.9	73.5
P3	7,344	99.9	73.4
CD44+CD24-	0	0.0	0.0
CD24+	0	0.0	0.0

Appendix Figure S3b

BD FACSDiva 8.0.1

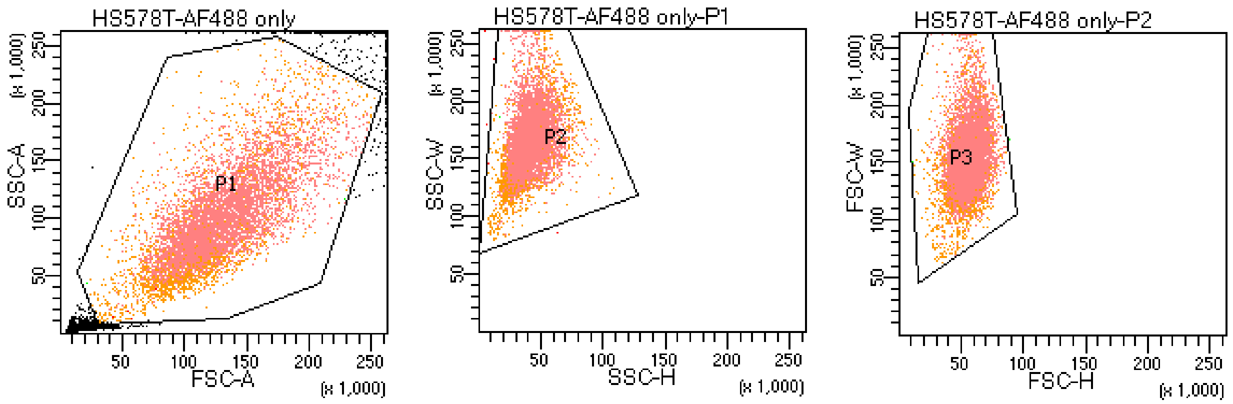

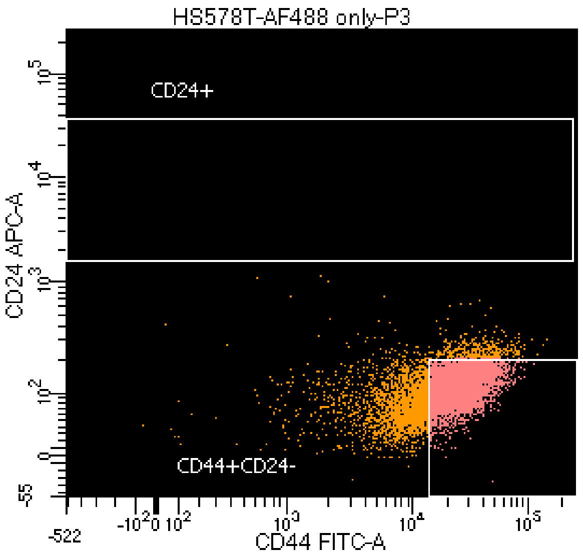

Tube: AF488 only

Population	#Events	%Parent	%Total
All Events	10,000	####	100.0
P1	7,867	78.7	78.7
P2	7,862	99.9	78.6
P3	7,860	100.0	78.6
CD44+CD24-	5,050	64.2	50.5
CD24+	0	0.0	0.0

Appendix Figure S3c

BD FACSDiva 8.0.1

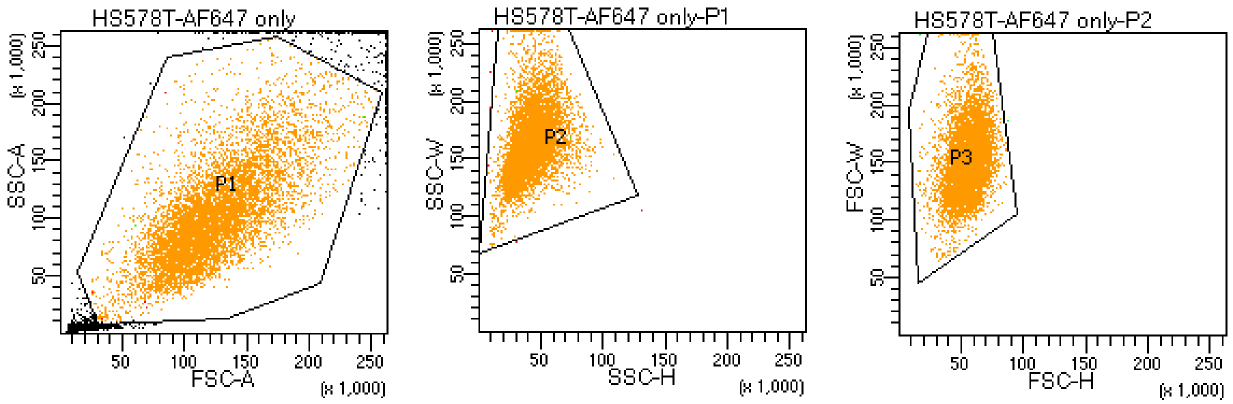

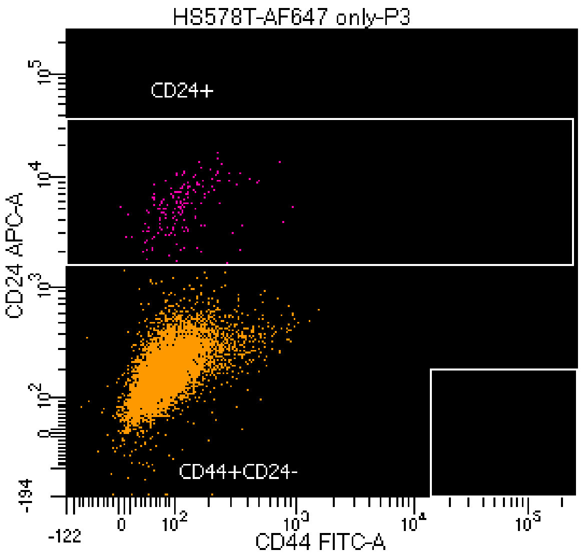

Tube: AF647 only

Population	#Events	%Parent	%Total
All Events	9,812	####	100.0
P1	7,853	80.0	80.0
P2	7,846	99.9	80.0
P3	7,843	100.0	79.9
CD44+CD24-	0	0.0	0.0
CD24+	171	2.2	1.7

Appendix Figure S3d

BD FACSDiva 8.0.1

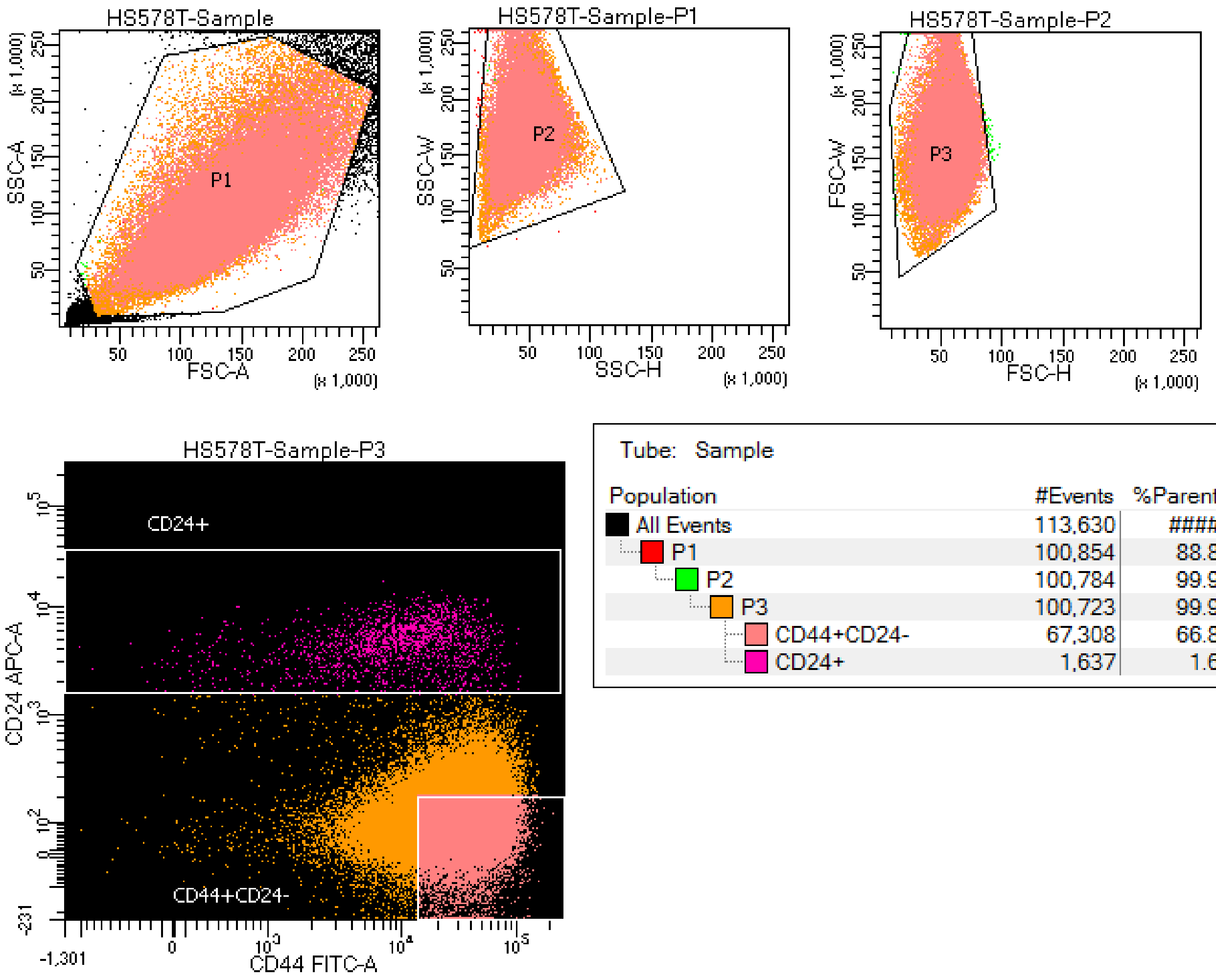

Tube: Sample

Population	#Events	%Parent	%Total
All Events	113,630	####	100.0
P1	100,854	88.8	88.8
P2	100,784	99.9	88.7
P3	100,723	99.9	88.6
CD44+CD24-	67,308	66.8	59.2
CD24+	1,637	1.6	1.4
