# Supplementary material for: Dynamic expression of tRNA‐derived small RNAs define cellular states
Source: EMBO Rep. 2019 Jun 12;20(7):e47789. doi: 10.15252/embr.201947789 (PMC6607006; doi:10.15252/embr.201947789)
Supplement: Supplementary file 2 — Expanded View Figures PDF [file EMBR-20-e47789-s002.pdf]

Expanded View Figures

Figure EV1. Profiling and characterisation of small RNAs and tsRNAs in heterologous cell and tissue models of stem vs. differentiated states.

A Expression of pluripotency genes in mESCs grown in stem or differentiating conditions (n = 2, error bars represent SD, and significance was calculated using Student's t-test).

B Size distribution of the sequence reads from small RNA libraries that mapped to the genome.

C Size distribution of the small RNA reads that map to the tRNA.

D Correlation of tRNA gene copy number and tsRNA abundance. tRNA gene copy numbers for every anticodon are represented as percentages of the total tRNA copies on the genome.

E Comparison of small RNA population changes in RA-induced differentiating cells treated with T4 PNK.

F tsRNA profile of bulge stem cell ($CD34^{+}\alpha6^{+}$) or basal cell ($CD34^{-}\alpha6^{+}$) populations isolated from adult murine skin (see Appendix Fig S1).

G Images of HMECs (Panels I-IV) engineered to represent stages of oncogenic transformation: I: parental HMECs immortalized with hTERT; II: immortalized HMECs with tamoxifen-inducible oncogenic HRAS (hTERT/sT/HRASG12V:ER/EV); III: HMEC as described in II with additional p53 knockdown (hTERT/sT/HRASG12V:ER/shP53); HMECs I-III were treated with 130 nM tamoxifen (5 days); and IV: HMEC II treated with tamoxifen (10 days).

G′ Quantification of 30–35 nt RNA levels in HMECs I–IV (n = 3, error bars represent SD, and significance was calculated using Student's t-test).

H, I tsRNA profiles of stem ($CD44^{+}CD24^{-}$) vs. differentiated ($CD24^{+}$) populations sorted from breast cancer cell lines: MDA-MB-231 (H) and HS578T (I) (see Appendix Figs S2 and S3).

J Heat map of tsRNA reads mapped to parent tRNA residues across various samples sequenced in this study. Majority of the tsRNAs identified were processed specifically from the 5′-half of the mature tRNA.

Data information: *P value < 0.05, **P value < 0.01; ***P value < 0.001; n.s, non-significant.

© 2019 The Authors

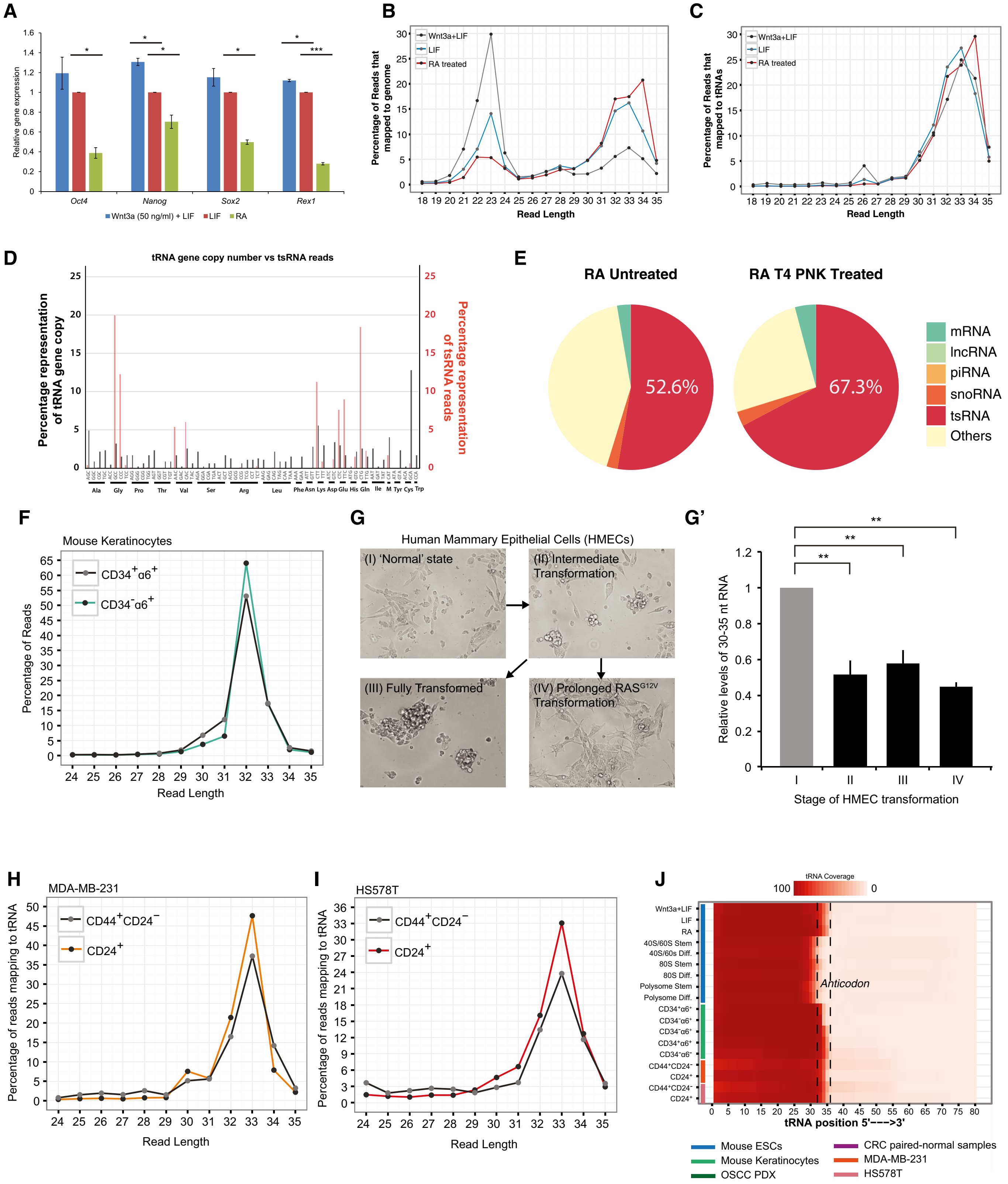

Figure EV1.

 © 2019 The Authors

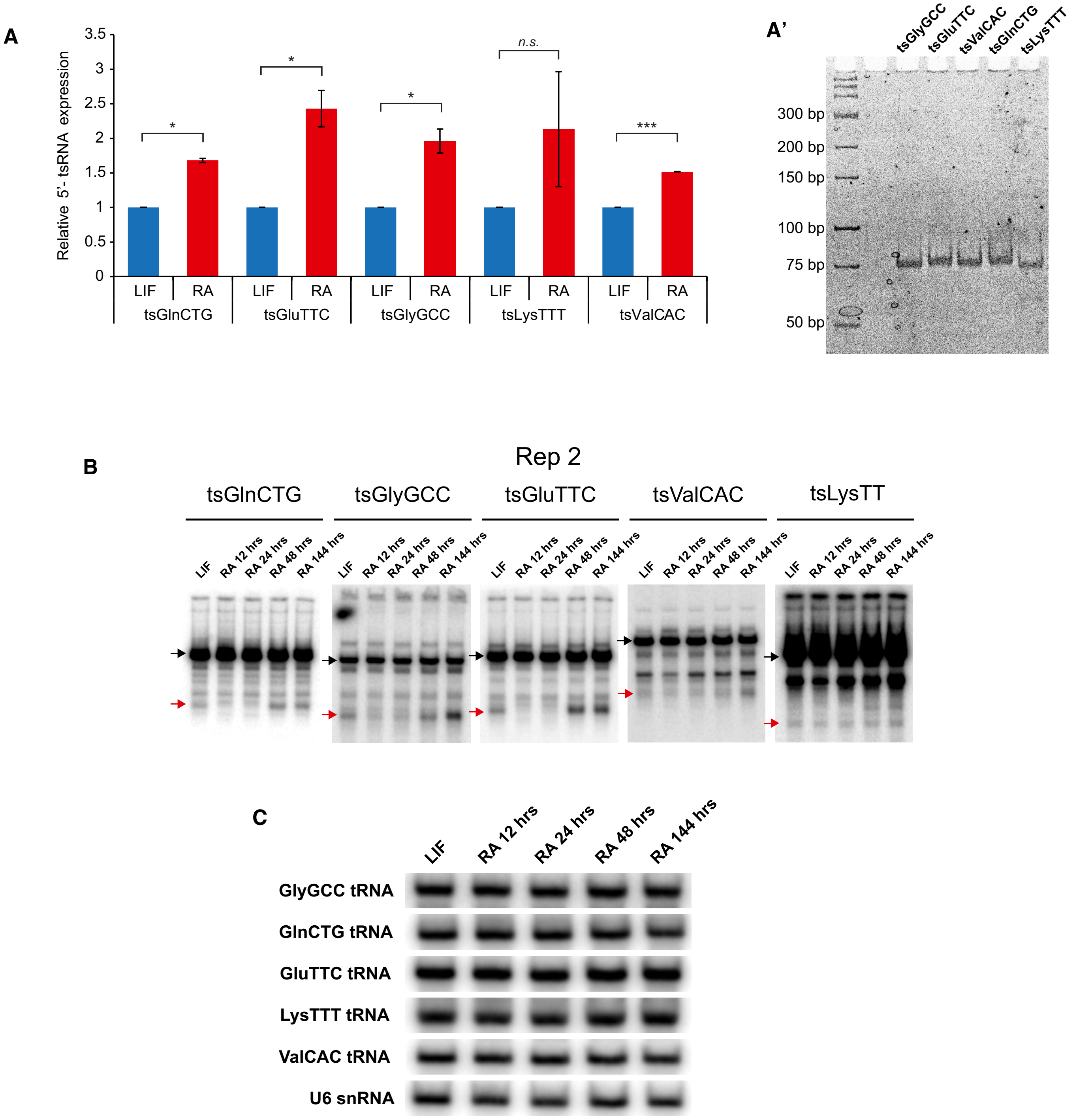

Figure EV2. Northern blot and qPCR validation of tsRNA expression in LIF and RA treated mESCs.

A qPCR validation of tsRNAs between LIF and RA at 48 h of differentiation ($n = 2$, error bars represent SD, and significance was calculated using Student's t-test).

A′ Gel showing the tsRNA band amplified in the qPCRs.

B Replicate of northern blots of candidate tRNA and 5′-tsRNAs across time points of RA differentiation. Black arrows indicate tRNA species, and the red arrows indicate tsRNA species.

C Northern blot of parent tRNA across various time points of RA-induced differentiation.

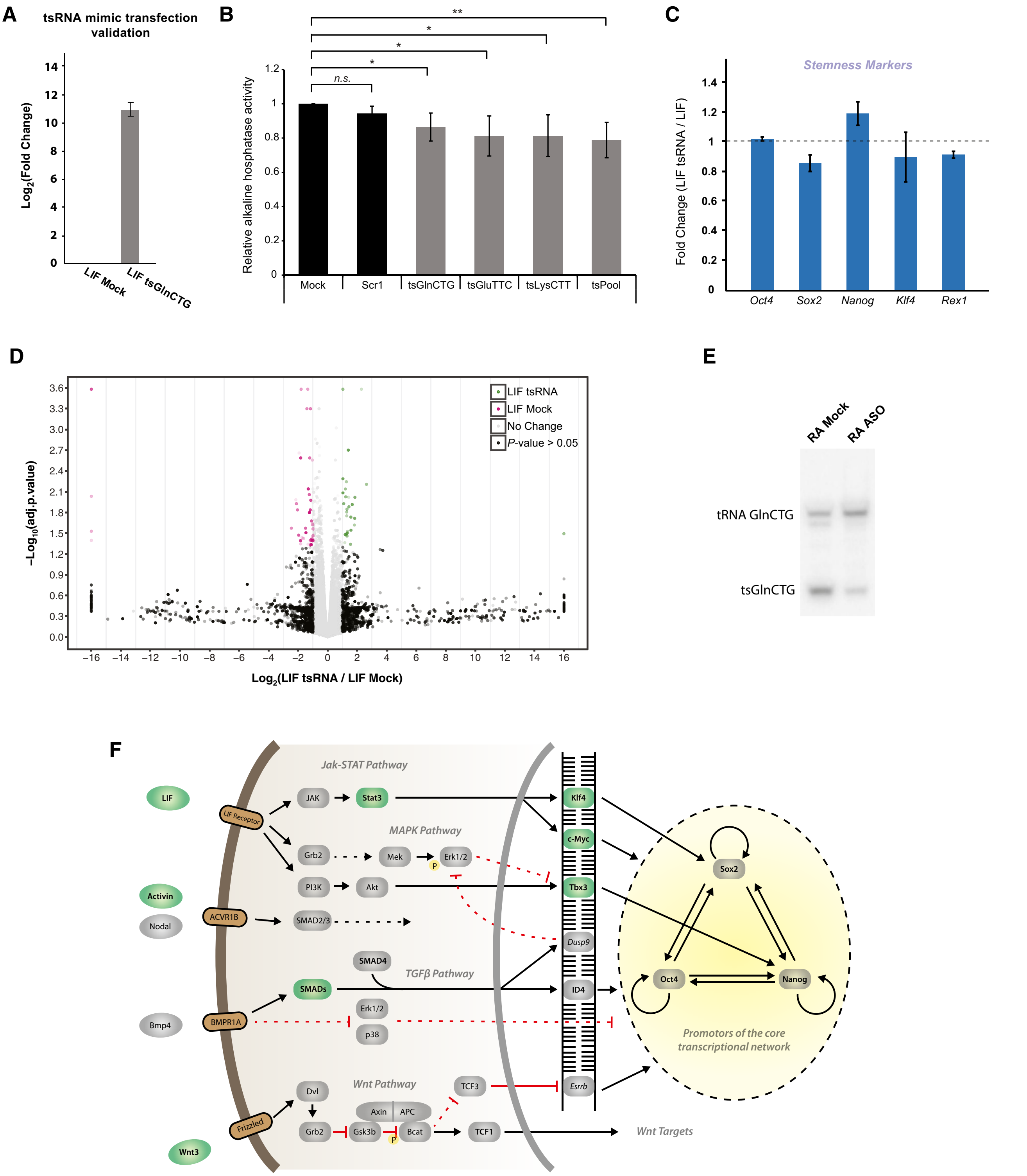

Figure EV3. Functional interrogation of tsRNAs in the modulation of stem and differentiated states in mESCs.

A qPCR quantitation of tsGlnCTG, transfected in LIF conditions (n = 3, error bar represent SEM, and significance was calculated using unpaired t-test).
B Alkaline phosphatase assays of tsRNA mimic overexpression in LIF condition (n = 3, error bar represent SD, and significance was calculated using unpaired t-test).
C Relative expression of stemness marker in tsRNA transfected in LIF conditions (n = 2, error bars represent SEM, and significance was calculated using unpaired t-test).
D Volcano plot showing transcripts upregulated (in green) and downregulated (in pink) in LIF samples transfected with tsRNA mimics compared with mock controls.
E Northern blot of tsGlnCTG in RA- vs. ASO-transfected RA cells.
F Components of various stemness pathways upregulated (in green) in ASO-treated RA conditions.

Data information: **P* value < 0.05, ****P* value < 0.001, *****P* value < 0.0001.

 © 2019 The Authors

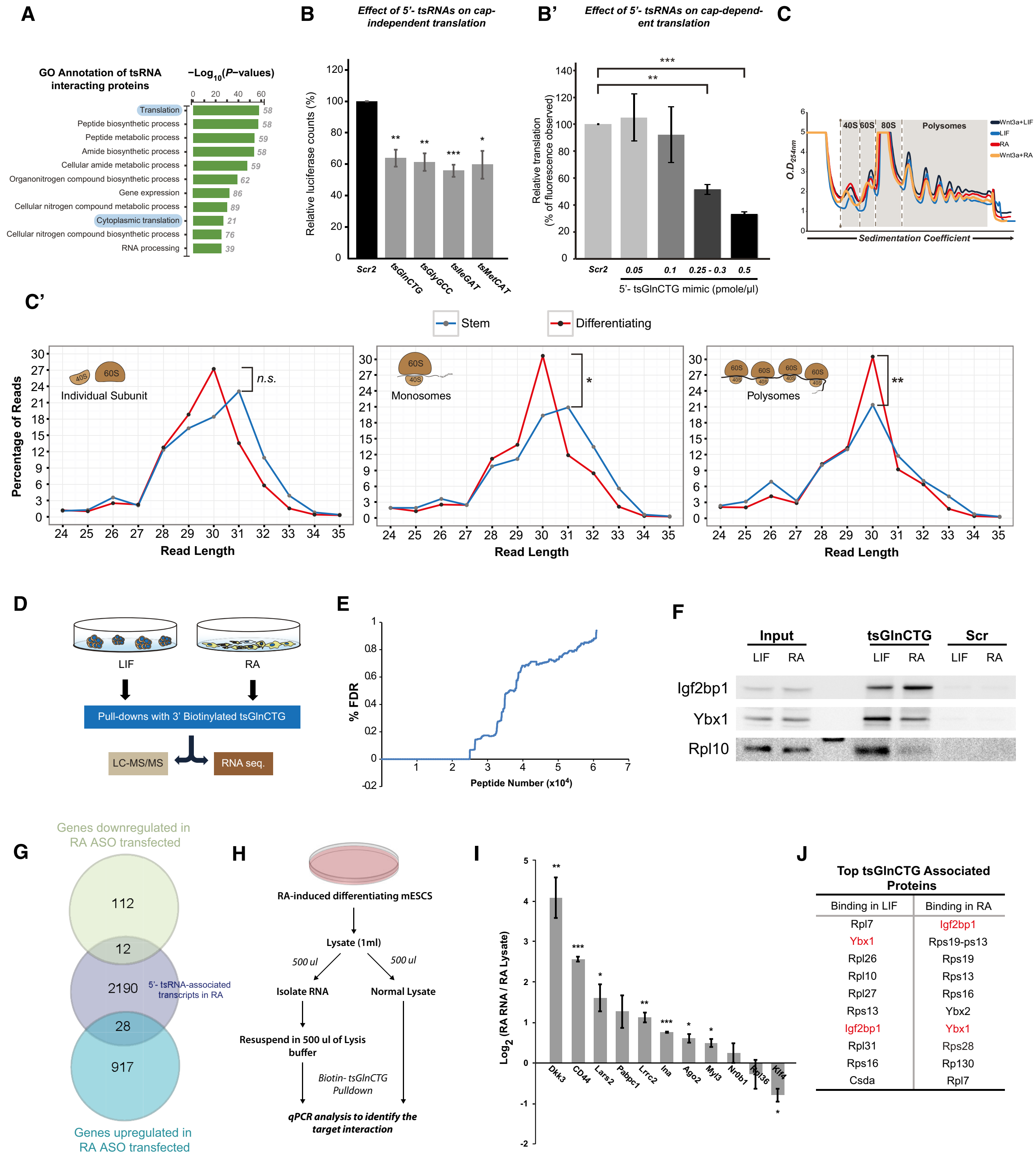

Binding in LIF	Binding in RA
Rpl7	Igf2bp1
Ybx1	Rps19-ps13
Rpl26	Rps19
Rpl10	Rps13
Rpl27	Rps16
Rps13	Ybx2
Igf2bp1	Ybx1
Rpl31	Rps28
Rps16	Rp130
Csda	Rpl7

Figure EV4.

Figure EV4. Characterization of mechanism and molecular function of candidate tsRNAs in pluripotent vs. differentiating mESCs.

A GO annotation of 109 common tsRNA-interacting proteins.

B Effects of candidate 5′-tsRNAs: tsGlnCTG, tsGlyGCC, tsMetCAT, and tsIleGAT on uncapped luciferase mRNA in an *in vitro* translation system (n = 2–3, error bars represent SEM, and significance was calculated using unpaired t-test).

B′ Effects of tsGlnCTG on capped GFP mRNA in an *in vitro* translation system (n = 2–3, error bars represent SD, and significance was calculated using unpaired t-test).

C Polysome profiles of different stem vs. differentiating states tested in this study. Gray areas indicate the fractions collected for sequencing.

C′ tsRNA distribution among the polysome fractions from indicated cell states.

D Workflow to identify proteomic and transcriptomic interactions of tsGlnCTG in pluripotency vs. differentiating mESCs.

E False discovery rate (FDR) of peptides detected in LC-MS/MS of biotinylated tsGlnCTG pulldown.

F Western blot showing differential interaction of Igf2bp1, Ybx1, and Rpl10 with tsGlnCTG between LIF and RA conditions.

G Overlap between transcripts that are associated with tsRNAs in RA condition and the transcripts that are affected upon ASO transfection in RA conditions.

H Schematic of the experimental setup to identify protein-mediated and complementarity-based tsRNA target interaction.

I qPCR analysis showing the enrichment of transcripts pulled down by tsGlnCTG in lysates devoid of proteins compared with normal lysate (n = 2, error bars represent SEM, and significance was calculated using unpaired t-test).

J Top 10 tsGlnCTG-associated proteins in indicated cell states.

Data information: *P value $<$ 0.05, **P value $<$ 0.01; ***P value $<$ 0.001; n.s, non-significant.

 © 2019 The Authors

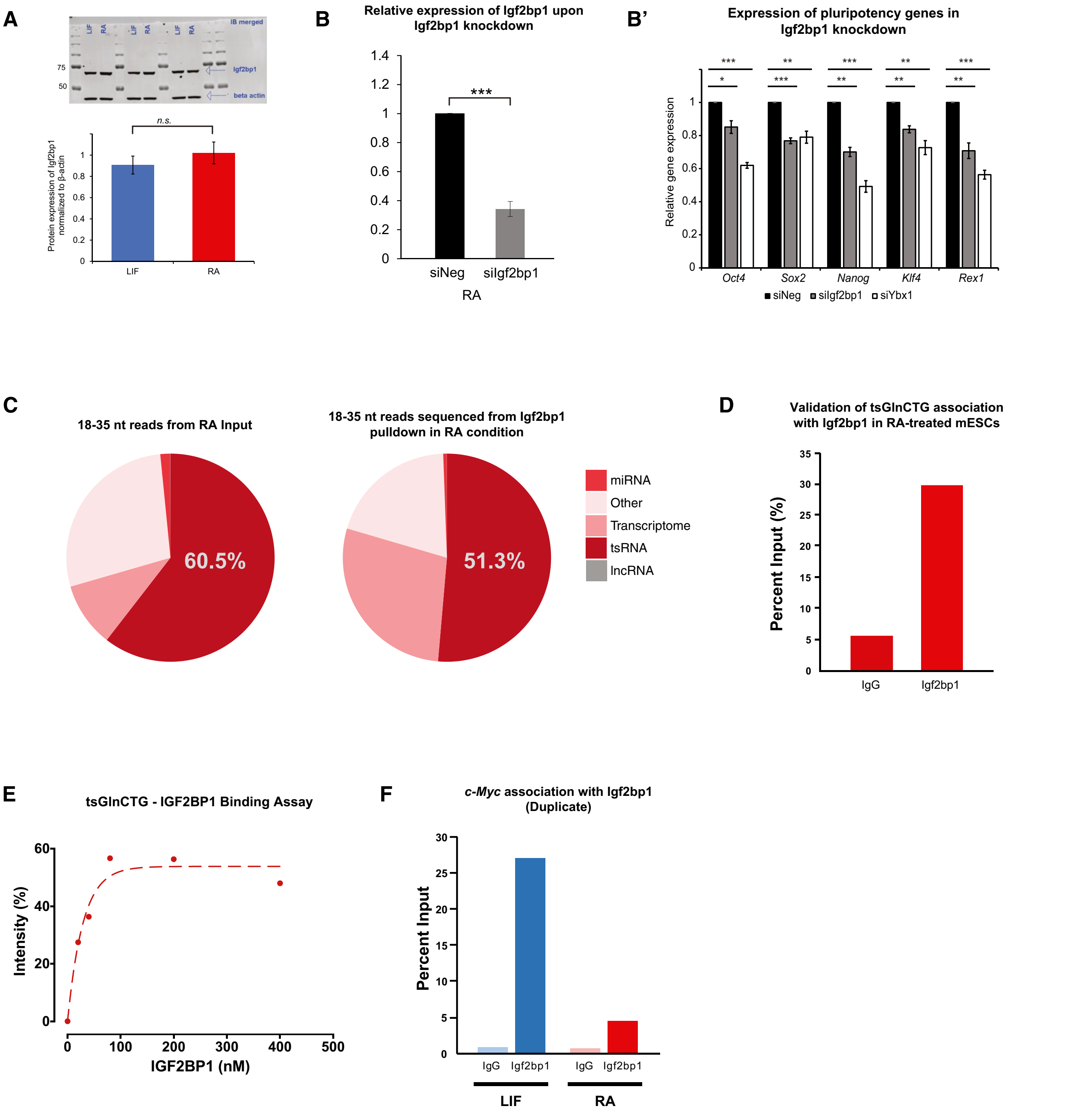

Figure EV5. Functional characterization of tsGlnCTG in the regulation of IGF2BP1 and c-Myc.

A Merged triplicate Western immunoblots of Igf2bp1 and β-actin in mESCs grown under LIF or RA conditions (top) and densitometric quantitation of the same (below) (n = 3, error bars represent SD, and significance was calculated using Student's t-test).

B Validation of siRNA-mediated knockdowns of Igf2bp1 in RA-treated mESCs (n = 3, error bars represent SD, and significance was calculated using Student's t-test).

B′ Expression of pluripotency genes in Igf2bp1 knockdown cells (n = 3, error bars represent SD, and significance was calculated using Student's t-test).

C Small RNAs identified (18–35 nt) from input and Igf2bp1 pulldown in RA-treated mESCs.

D qPCR validation of the tsGlnCTG enrichment upon Igf2bp1 pulldown in RA-treated mESCs.

E Binding curve showing the interaction of tsGlnCTG and Igf2bp1 (Kd = 33 nM). The intensities of the Igf2bp1 band were normalized to band intensity of the total Igf2bp1 protein used for pulldown.

F Biological duplicate for c-Myc quantitation from Igf2bp1 pulldown between LIF vs. RA conditions (n = 2, for duplicate check Fig 5B).

Data information: *P value < 0.05, **P value < 0.01; ***P value < 0.001; n.s, non-significant.

© 2019 The Authors
